# Supplementary figures and images for: Improving ancient DNA genome assembly
Source: PeerJ. 2017 Apr 5;5:e3126. doi: 10.7717/peerj.3126 (PMC5384568; doi:10.7717/peerj.3126)

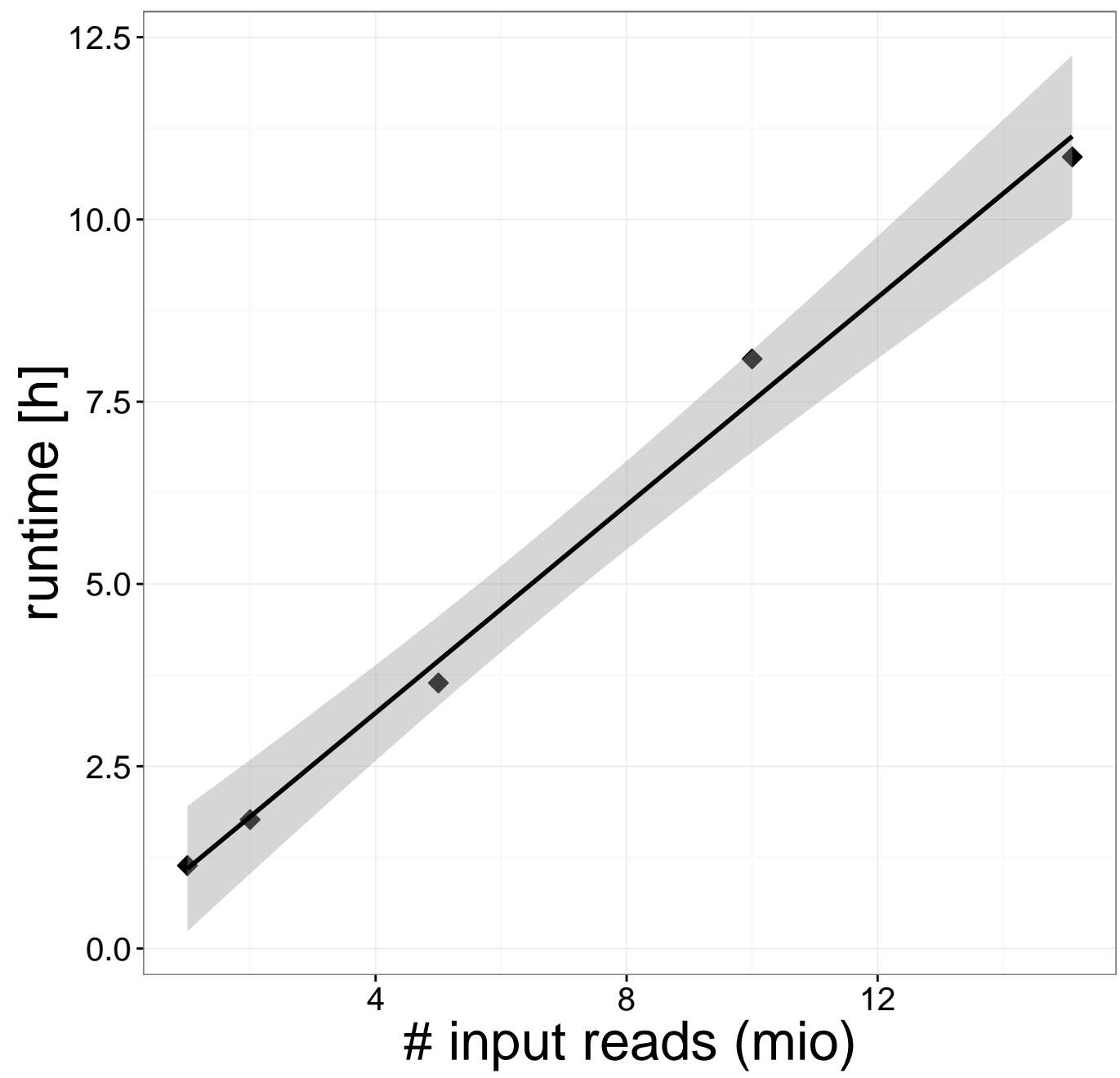

Supplement: Figure S1 — The dots show the time in min using 1,2,5,10,15.1 mio input reads. The black line shows the linear regression and the grey area depicts the 95% confidence region. [file peerj-05-3126-s002.pdf]
